# The dual PI3K/mTOR inhibitor dactolisib elicits anti-tumor activity *in vitro* and *in vivo*

**DOI:** 10.18632/oncotarget.23091

**Published:** 2017-12-09

**Authors:** Fei Shi, Jinying Zhang, Hongyu Liu, Liangliang Wu, Hongyu Jiang, Qiyan Wu, Tianyi Liu, Meiqing Lou, Hao Wu

**Affiliations:** ^1^ Department of Neurosurgery, Shanghai General Hospital, Shanghai Jiao Tong University School of Medicine, Shanghai 200000, China; ^2^ Institute of Basic Medicine Science, Chinese PLA General Hospital, Beijing 100853, China; ^3^ Key Laboratory of Cancer Center, Chinese PLA General Hospital, Beijing 100853, China; ^4^ Department of Anesthesiology, Wuxi Third People’s Hospital, Wuxi, Jiangsu 214000, China; ^5^ Department of Neurosurgery, Xuanwu Hospital, Capital Medical University, Beijing 100053, China

**Keywords:** chemotherapy, dactolisib (NVP-BEZ235), dual PI3K/mTOR inhibitor, glioblastoma, radiotherapy

## Abstract

Glioblastomas (GBMs) are among the most malignant of all human tumors and have poor prognosis. The current standard of care (SOC) includes maximal surgical tumor resection followed by adjuvant temozolomide (TMZ) and concomitant radiotherapy (RT). However, even with this treatment, the 5-year survival rate is less than 10%, and thus, follow-up treatment is required to improve efficacy. In GBMs as well as many other solid cancers, PI3K/mTOR signaling is overactivated. Therefore, multiple tumor-based PI3K inhibitors have been studied in various cancers. In the current study, we investigated the effect of the dual PI3K/mTOR inhibitor dactolisib on TMZ+RT treatment in three human GBM cell lines and a orthotopic xenograft model. Dactolisib alone induced cytotoxicity and pro-apoptotic effects, which act as antitumor factors. Combined with SOC treatment, dactolisib inhibited cell viability, induced enhanced pro-apoptotic effect, and attenuated migration/invasion in all three cell lines, thereby enhancing the SOC therapeutic effect. Protein microarray analysis showed that A172 cells treated with TMZ+RT+dactolisib had higher p27 and lower Bcl-2 expression than other groups. Moreover, in the xenograft model, oral dactolisib combined with TMZ+RT inhibited tumor growth and prolonged survival. Thus, SOC combined with dactolisib shows potent anti-tumor activity and has promising potential for solid tumor treatment.

## INTRODUCTION

Glioblastoma multiforme (GBM) is a very common malignant tumor in the brain and spinal cord. It is usually leads to death, with a mean survival time of 8–12 months [1]. Currently, the standard of care (SOC) for malignant glioma is maximal surgical tumor resection followed by adjuvant temozolomide (TMZ) and radiotherapy (RT). TMZ is an antitumor agent with good blood-brain barrier (BBB) penetration [2] and is recommended in combination with RT. TMZ has a cytotoxic effect through methylating guanine in DNA. However, evidence shows that GBMs become easily resistant to alkylating agents such as TMZ [3]. In addition, the PI3K-AKT pathway can be activated and induce resistance to alkylating agents [4]. Moreover, because of the invasive growth of GBM, which often leads to incomplete resection, even with this treatment, the 5-year survival rate is less than 10% [5, 6]. Therefore, an improved treatment modality for GBM is imperative.

The activity of the phosphatidylinositol 3-kinase (PI3K)/mTOR signaling pathway is associated with cell motility, survival, and chemotherapy resistance. The PI3K/mTOR pathway is generally deregulated in cerebral and spinal malignant neoplasms [7] and is related to resistance to TMZ [8]. The inhibition of signaling pathways is a promising approach for the treatment of neurological malignancies, as most patient with malignant tumors show pathway overactivation [9]. Consequently, an array of tumor-based PI3K inhibitors have been studied in various cancers, including melanoma, glioma, hepatocellular carcinoma, and renal cell carcinoma. Some inhibitors of the pan-PI3K or PI3K/mTOR pathway have been shown to enhance the effect of SOC therapy [10, 11]. However, some inhibitors, such as LY294002, have limited clinical potential because of high cytotoxicity [12, 13].

To overcome such clinical concerns, we are studying the application potential of the new and highly specific PI3K/mTOR inhibitor, dactolisib (NVP-BEZ235), in GBM [14, 15]. Dactolisib, an orally administered potent dual inhibitor of PI3K/mTOR, indicates promising anti-solid tumor efficacy [15–17]. For example, dactolisib has exhibited promising anti-neoplastic activity in prostate cancer [15]. While the BBB is often an important obstacle to brain tumors, it is commonly disrupted in high-grade brain tumors and after surgery [18–20]. Therefore, it can be assumed that dactolisib has good penetration ability in high-grade GBM after surgery. Previous studies have shown that an appropriate dactolisib concentration can indeed cross the BBB [17, 21]. However, in some clinical trials in certain tumors, patients were found to suffer side effects, and we have observed side effects, including rash and hair loss, in small number of rats in a previous study [22].

Here, we present evidence that in human GBMs, the dual PI3K/mTOR inhibitor dactolisib inhibits cell viability, induces apoptosis, and enhances the RT+TMZ treatment effect in glioma cell lines. Microarray analysis was used to confirm these changes at the protein level in A172 GBM cells. We showed that dactolisib suppresses p-AKT and mTOR expression and promotes p27 expression, while reducing Bcl-2 levels. Finally, this study provided the first preclinical evidence that dactolisib enhances the TMZ+RT effect, in an orthotopic xenograft rat model.

## RESULTS

### Dactolisib enhances the effect of TMZ+RT in reducing GBM cell viability

**Figure 1:**
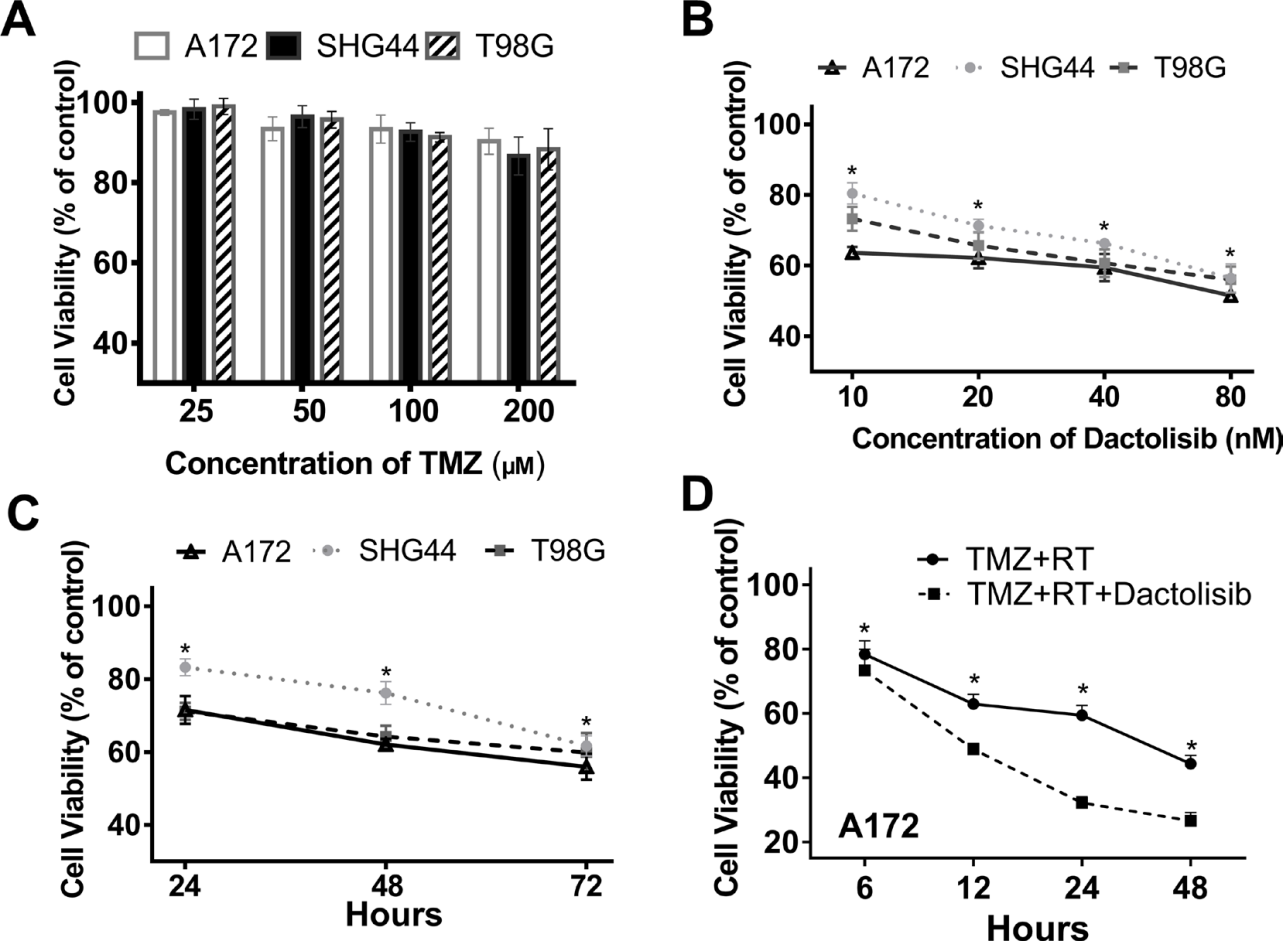
Dactolisib treatment enhances the reduction in GBM cell viability by TMZ+RT (**A**) Cell viability in A172, SHG44, and T98G GBM cells treated with 25–200 μM TMZ for 24 h. (**B**) Cell viability in A172, SHG44, and T98G GBM cells treated with 10–80 nM dactolisib. Cell viability was assessed after 48 h of drug exposure. *P < 0.05 vs. control. (**C**) Cell viability in A172, SHG44, and T98G GBM cells treated with 20 nM dactolisib for 24, 72, and 48 h. (**D**) Cell viability in A172 cells exposed to TMZ+RT or TMZ+RT+dactolisib for 48 h. *P < 0.05 vs. control. DMSO served as a vehicle control. Cell viabilities were assessed by CCK-8. Treatments included TMZ (100 μM), RT (2 Gy), and dactolisib (20 nM).

The effects of TMZ, dactolisib, TMZ+RT, and TMZ+RT+dactolisib on cell viability were evaluated in A172, SHG44, and T98G GBM cells by CCK-8 assay. Dimethyl sulfoxide (DMSO) was used as a control treatment. CCK8 assay revealed that TMZ (100 µM) alone had a limited effect on GBM cell viability (Figure 1A). Dactolisib showed a significant, dose-dependent effect in SHG44 and T98G cells as compared to the control, while in A172 cells, the treatment efficacy showed only a moderate increase with a four-fold increase in dose (10 nM vs. 40 nM) (Figure 1B). Further, dactolisib treatment showed a time-dependent effect in the three tested lines (Figure 1C). Dactolisib strongly enhanced the inhibitory effect of TMZ+RT on cell viability in A172 cells (Figure 1D).

### Dactolisib enhances the effect of TMZ+RT in suppressing GBM cell migration and invasion

**Figure 2:**
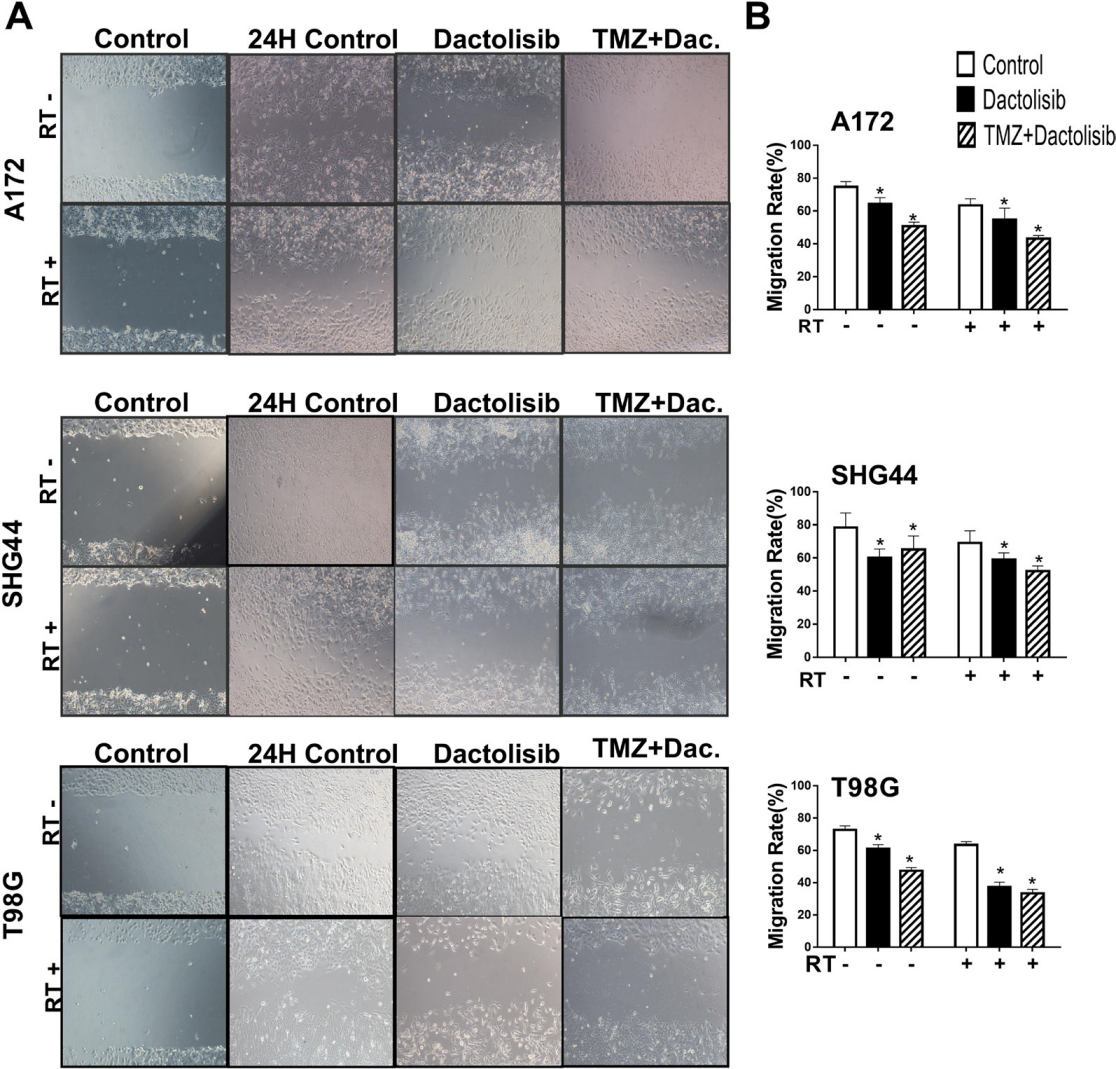
Dactolisib enhances the inhibitory effect of TMZ+RT on migration of glioma cells (**A**) Micrographs showing cell migration of A172, SHG44, and T98G cells exposed to dactolisib, TMZ+dactolisib, or TMZ+RT+dactolisib for 24 hours. (**B**) Quantitative data of cell migration as shown in panel (A) Data are from three independent experiments. *P < 0.05 vs. control. Treatments included TMZ (100 μM), RT (2 Gy), and dactolisib (20 nM).

**Figure 3:**
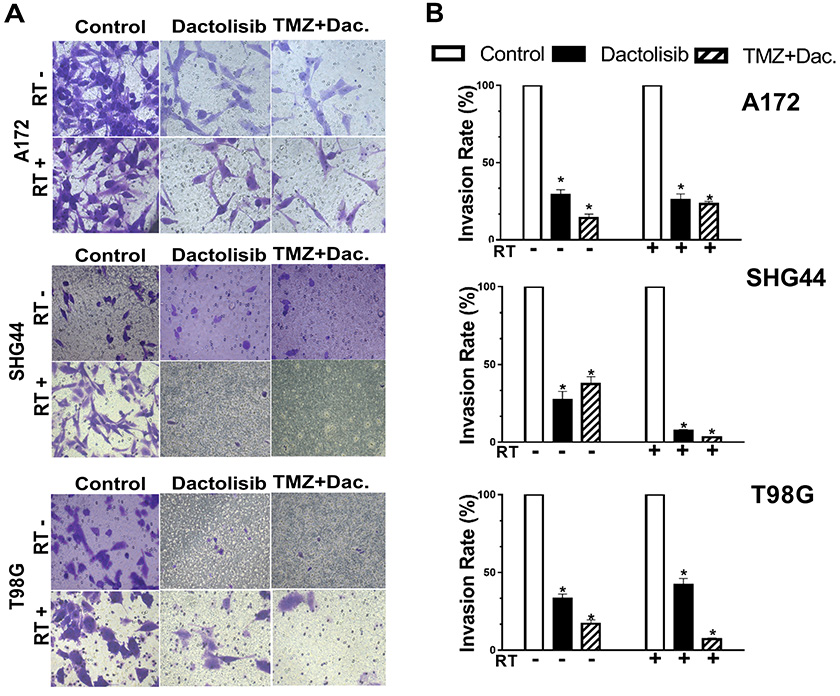
Dactolisib enhances the inhibitory effect of TMZ+RT on invasion of glioma cells (**A**) Micrographs showing cell invasion of A172, SHG44, and T98G cells exposed to dactolisib, TMZ+dactolisib, or TMZ+RT+dactolisib for 24 hours. (**B**) Quantitative data of cell invasion as shown in panel (A). Data are from three independent experiments. *p < 0.05 from control. Treatments included TMZ (100 μM), RT (2 Gy), and dactolisib (20 nM).

The effects of dactolisib, TMZ+dactolisib, and TMZ+RT+dactolisib on cell migration and invasion of A172, SHG44, and T98G GBM cells were evaluated by wound healing (Figure 2) and invasion assays (Figure 3), respectively. In the presence of dactolisib, RT+TMZ significantly inhibited cell migration/invasion in all cell lines tested, while TMZ combined with RT, had no significant inhibition of migration and invasion (Figures 2B and 3B). And dactolisib alone implied a better results than control groups in different three cell lines.

### Dactolisib enhances the pro-apoptotic effects of RT+TMZ in GBM cells

**Figure 4:**
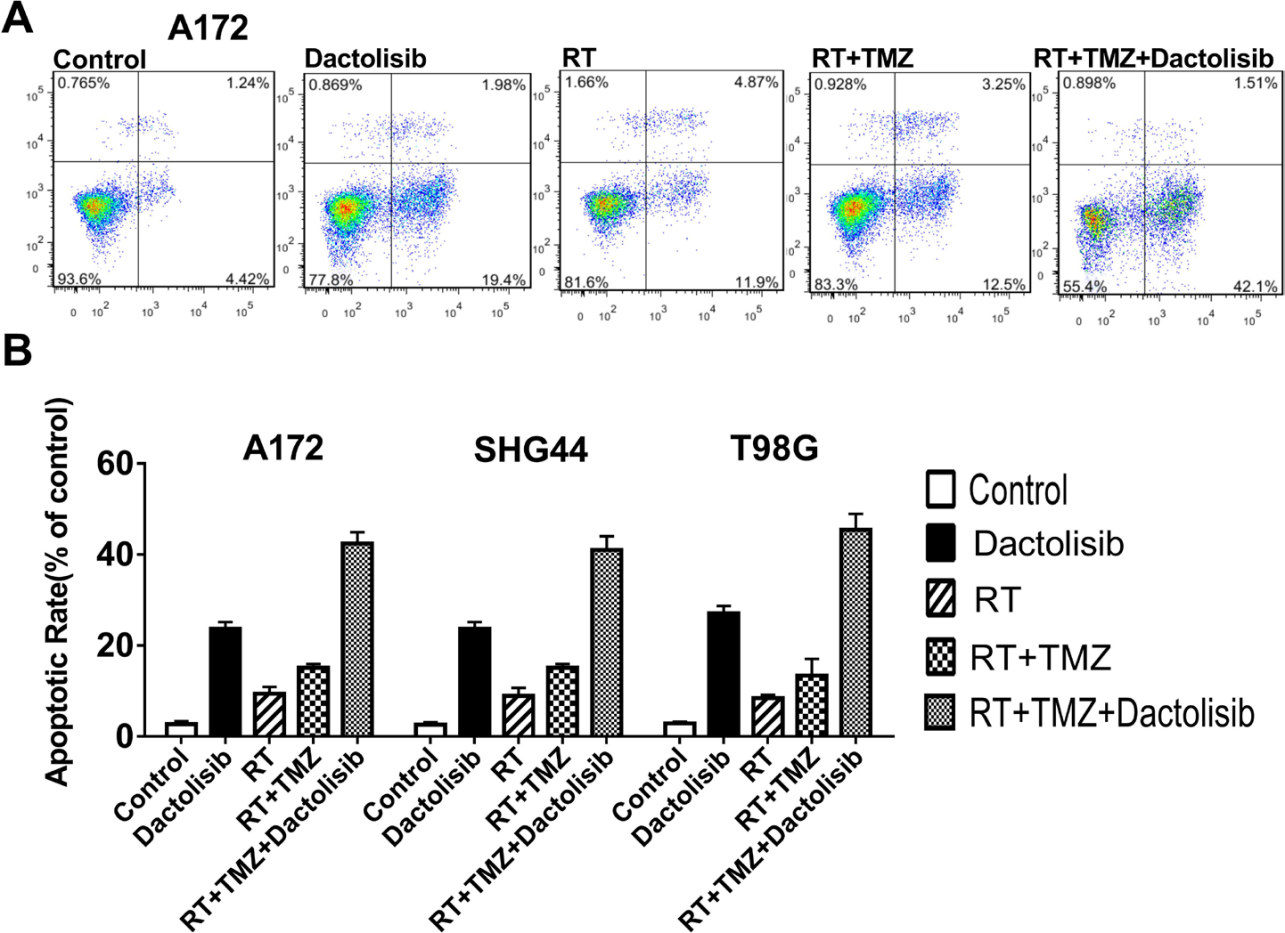
Dactolisib enhances the pro-apoptotic effect of TMZ+RT in glioma cells (**A**) Representative flow-cytometric results of the effects of dactolisib, RT, RT+TMZ, and TMZ+RT+dactolisib in A172 cells. Cells were treated for 24 hours. (**B**) Histograms showing the percentage of apoptotic cells in A172, SHG44, and T98G cells treated with dactolisib, RT, RT+TMZ, and TMZ+RT+dactolisib. Data are from three independent experiments. Treatments included TMZ (100 μM), RT (2 Gy), and dactolisib (20 nM).

Pro-apoptotic effects of the various treatments were studied using flow cytometry with Annexin V-FITC/PI staining. Annexin V-FITC+ cells represent apoptotic cells, whereas necrotic cells are V-FITC+/PI+. After treatment with 20 nM dactolisib+100 μM TMZ+RT for 24 h, 44.5 **±** 3.5% of A172 cells were apoptotic. After 24-h treatment in the absence of RT, 30.3 ± 1.9% of A172 cells were apoptotic. The apoptosis-inducing effect of TMZ+RT was significantly enhanced by treatment with dactolisib (Figure 4). Similar results were obtained in SHG44 and T98G cells (data not shown).

### Dactolisib changes the duration of phases of the cell cycle in GBM cell lines

**Figure 5:**
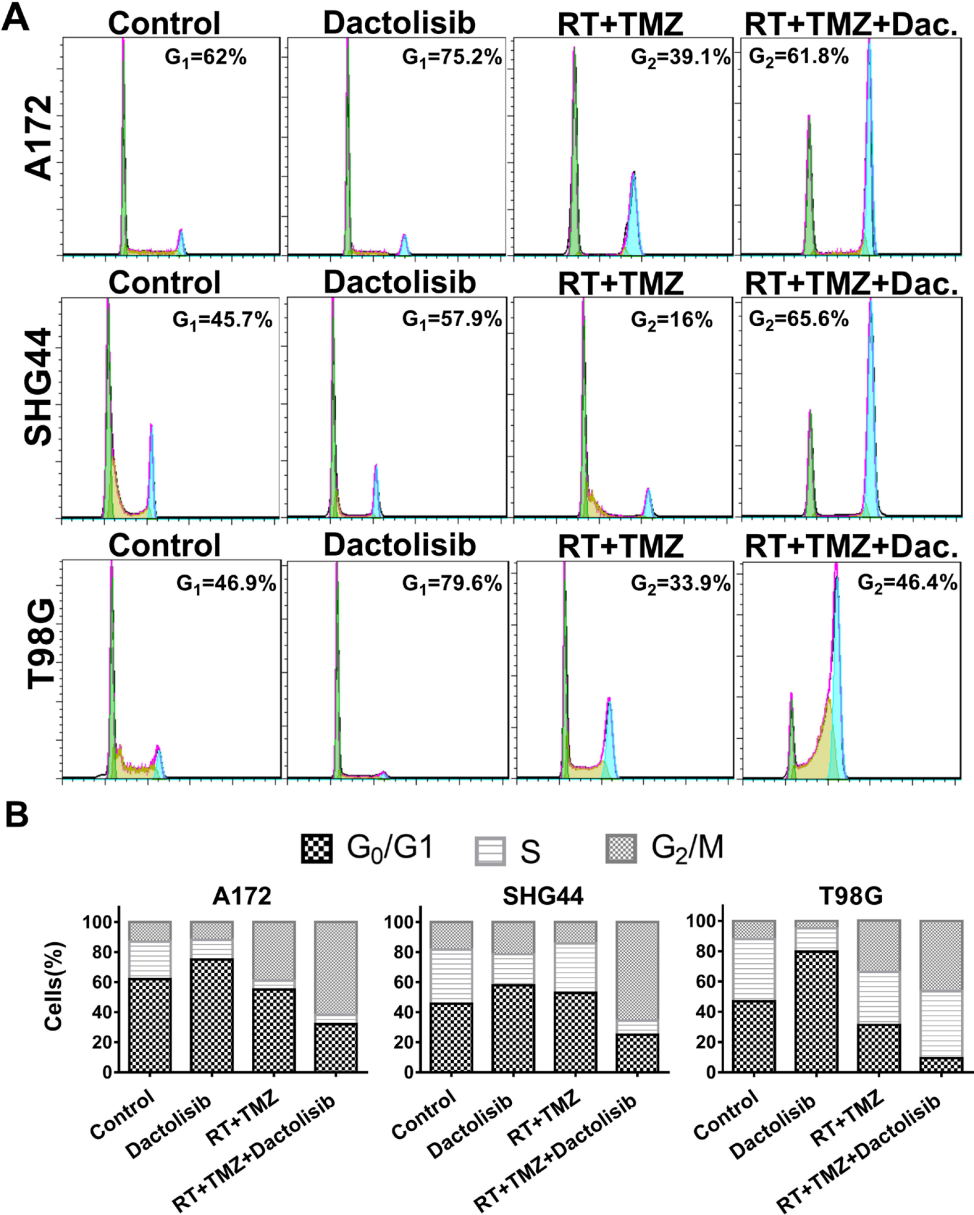
Treatment with TMZ, RT, and dactolisib induces cell-cycle arrest in glioma cells (**A**) Representative flow-cytometric results showing the percentages of cells in G1, S, or G2-M phase in T98G, SHG44, and A172 cells exposed to dactolisib, RT+TMZ, or TMZ+RT+dactolisib for 24 hours. (**B**) Histograms showing the percentages of glioma cells in G1/G0, S, and G2/M phases. Treatments included TMZ (100 μM), RT (2 Gy), and dactolisib (20 nM).

We studied whether the PI3K/mTOR inhibitor affects the cell cycle. Cell-cycle distribution was evaluated by flow cytometry in all three GBM cell lines after 24-h treatments. Dactolisib alone induced cell-cycle arrest at G0/G1 phase as compared to the non-treated control group (Figure 5A). The primary mechanism of action of RT is that it accelerates senescence or induces terminal growth stagnation in cancer cells. Dactolisib alone induced G1 stagnation, whereas TMZ alone had only a limited effect. However, when dactolisib was combined with TMZ+RT, G2/M stagnation was significantly prolonged (Figure 5B).

**Figure 6:**
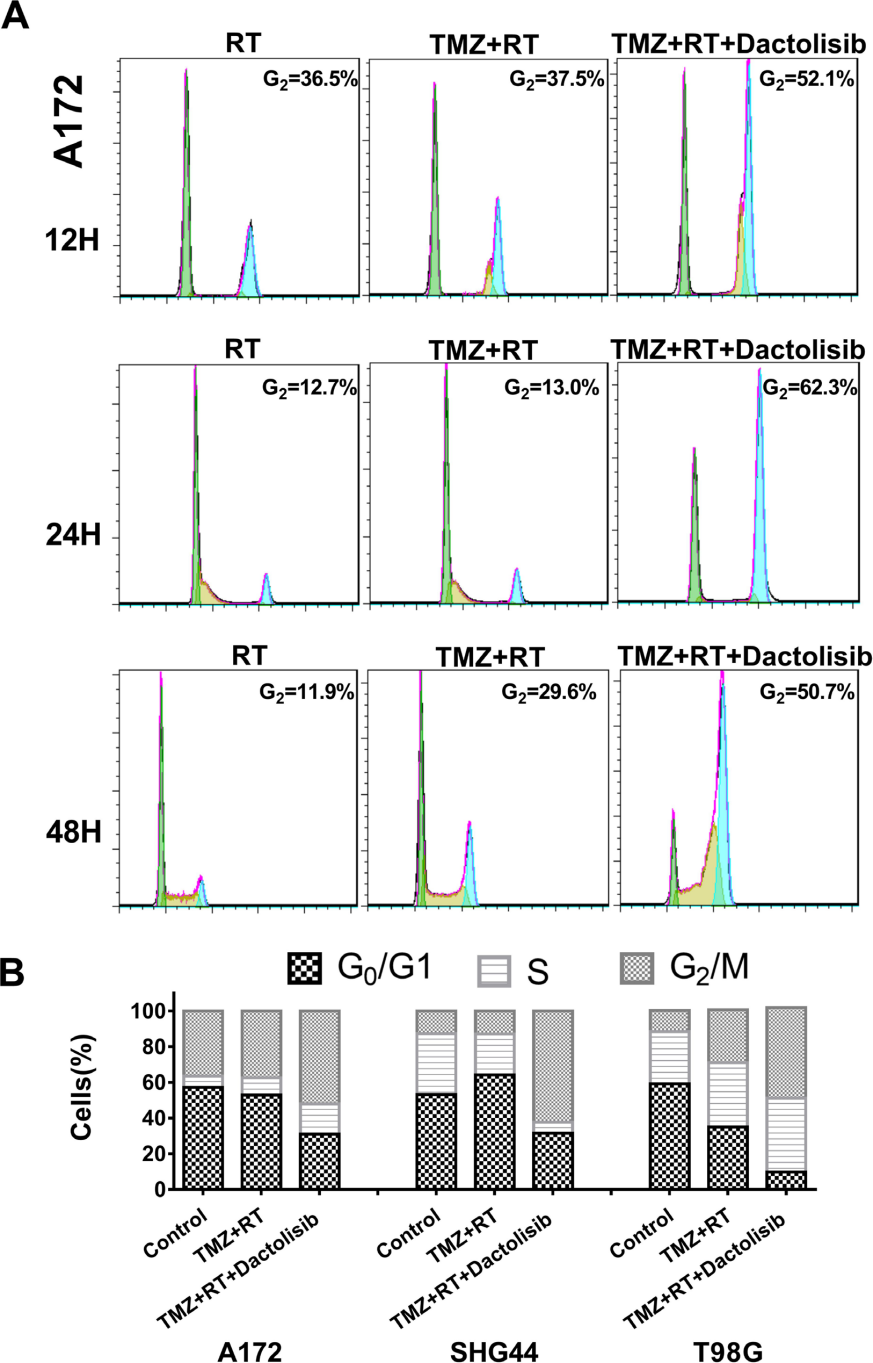
Cell-cycle arrest in glioma cells treated with TMZ, RT, and dactolisib in different time point (**A**) Representative flow-cytometric results showing the percentages of cells in G1, S, or G2-M phase in A172 cells after the indicated treatments for 12, 24, and 48 h. (**B**) Histograms showing the percentages of glioma cells in G1/G0, S, and G2/M phases. Treatments included TMZ (100 μM), RT (2 Gy), and dactolisib (20 nM).

In a time-course analysis of A172 cells treated for 12, 24 or 48 h, we observed a similar trend: dactolisib induced G1-phase cell-cycle arrest, whereas dactolisib induced G2-phase cell-cycle when combined with TMZ+RT treatment (Figure 6).

### Dactolisib acts via the PI3K/mTOR pathway

**Figure 7:**
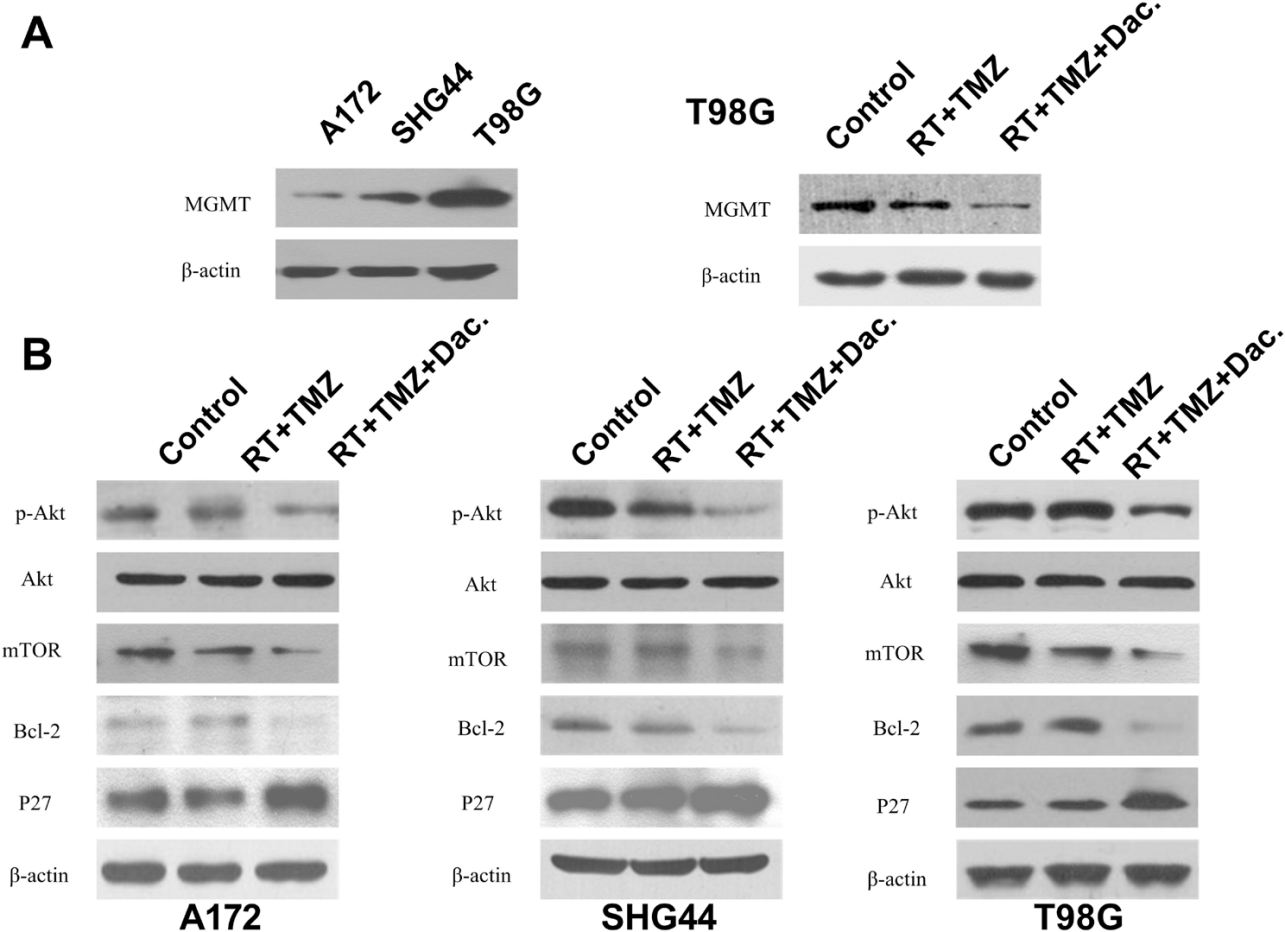
MGMT expression and dactolisib intervention on PI3K/mTOR pathway (**A**) O6-Methylguanine DNA methyltransferase (MGMT) expression was determined by western blot analysis. Data were obtained from three independent experiments. MGMT levels were reduced by dactolisib in T98G cells. (**B**) All three cell lines were treated with TMZ (100 μM), dactolisib (20 nM), RT (2 Gy), or the indicated combination for 24 h. Total protein level and phosphorylation status of AKT, Bcl-2, and p27 were determined by western blot analysis. β-Actin served as a loading control.

TMZ-induced damage can be repaired by the DNA repair enzyme, O6-methylguanine-DNA methyltransferase (MGMT), whose key role in TMZ resistance is now generally accepted [23]. T98G cells have been found to be resistant to TMZ [5]. Thus, we checked MGMT protein levels in these cells before and after the different interventions, using western blotting. In comparison to A172 and SHG44 cells, T98G cells indeed showed higher basal expression of MGMT (Figure 7A). While RT+TMZ suppressed MGMT protein expression, T98G cells exposed to TMZ+RT+dactolisib had even lower MGMT protein levels than cells exposed to RT+TMZ (Figure 7A).

To analyze whether the effect of TMZ+RT+dactolisib treatment is meditated by inhibition of the PI3K/mTOR pathway, we used an AKT/PKB Phosphorylation Antibody Array (PAB216) to detect differences in protein expression induced by TMZ+RT+dactolisib as compared to TMZ+RT in SHG44 cells (Supplementary Figure 1). We determined to be significantly differentially expressed based on the Phosphorylation Antibody Array analysis and therefore confirmed by western blotting. The total protein levels and the phosphorylation status of AKT, mTOR, p27, and Bcl-2 were determined by western blotting. Pretreatment with dactolisib resulted in a significant reduction in p-AKT and mTOR protein expression as compared to TMZ+RT treatment in these glioma cells (Figure 7B). Additionally, the antibody microarray assay revealed that p27 was upregulated by 39% **±** 3.4% in cells treated with TMZ+RT+dactolisib as compared to those treated with TMZ+RT. Bcl-2 was downregulated by 34% **±** 1.6% in the TMZ+RT+dactolisib group vs. the TMZ+RT group (Figure 7B). Similar results were obtained in A172 cells and T98G cells by western blot analysis (Figure 7B).

### Dactolisib delays GBM cell growth and prolongs survival of an orthotopic xenograft model

**Figure 8:**
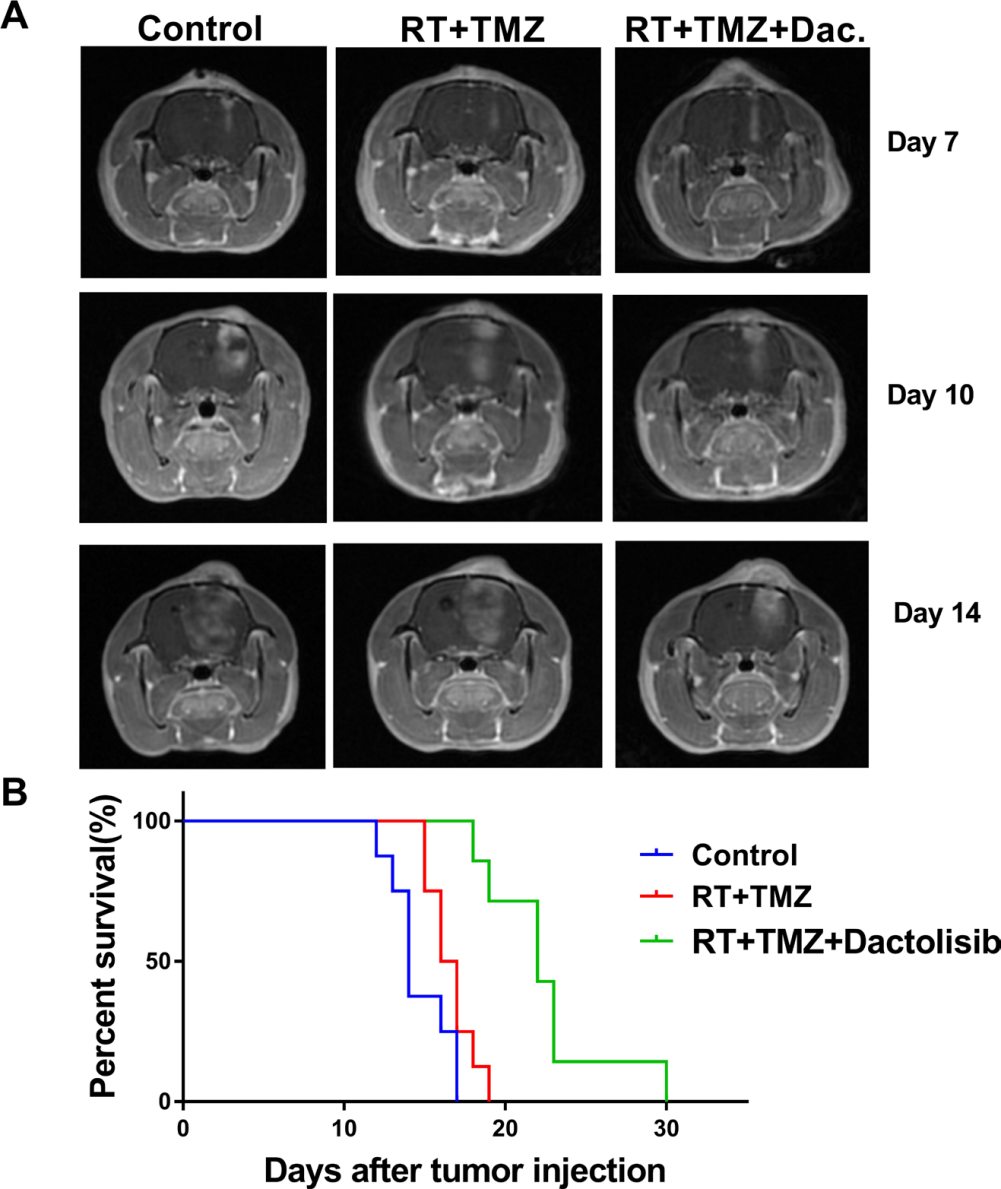
Dactolisib inhibits tumor growth *in vivo* and prolongs survival of orthotopic glioblastoma-bearing rats (**A**) Axial 3D fast spoiled gradient-echo images. Tumor volumes were significantly different among 3 groups on days 10 and 14. (**B**) Kaplan–Meier method was used to assess animal survival time. The survival time of rats in the RT+TMZ+dactolisib group was significantly longer than other groups.

To assess the *in-vivo* efficacy of dactolisib, an orthotopic GBM xenograft model was established (Figure 8A). To establish the model, SHG44 cells (2 **×** 10^5^) were injected into the right caudate nuclei of immunocompromised nude rats. Four days later, the rats were treated with TMZ+RT, TMZ+RT+dactolisib (20 mg/kg), or left untreated. Healthy immunocompromised nude rats exposed to dactolisib at a dose of 20 mg/kg do not show weight loss [28]. However, at a dose higher than 25 mg/kg, rats show enhanced side effects, including weight loss, rash, and hair loss. MRI confirmed tumor engraftment in all rats. Animals treated with dactolisib showed delayed occupying growth, which resulted in obviously longer survival compared to the control and RT+TMZ groups as indicated by the Kaplan–Meyer survival curves (Figure 8B).

## DISCUSSION

In this study, the use of dactolisib in combination with TMZ and RT was found to have a good therapeutic effect on the suppression of glioma growth. We found that 100 μM TMZ had little effect on inhibiting GBM cell viability, migration, and *in-vitro* invasion, while the combination of TMZ+RT+dactolisib yielded significantly better results. Our findings were consistent with previous study [24].

Treatment with RT is a better approach than other modalities for improving outcomes for some tumors. Radiotherapy (RT) of cancer cells induces cell death by different mechanisms. Mitotic catastrophe involves a series of events leading to cell death in the form of apoptosis, necrosis, or autophagy [25]. Radioresistance is associated with the level of AKT activation. Further, radiation can cause DNA damage, including double-strand breaks, which, if not repaired, results in G_2_/M arrest and finally, leads to cell death.

PI3K is involved in cell-cycle progression [26] and induces anti-proliferation and prolongs cancer-cell survival. Several studies have shown that PI3K inhibitors potently enhance the effects of RT and attenuate DNA repair after RT exposure [17, 27]. However, limited data are available on dactolisib-mediated enhancement of TMZ+RT effects. Therefore, this study aimed to investigate the effects of dactolisib in combination with TMZ+RT. We found that dactolisib alone induces G_0_/G_1_ phase arrest; however, when combined with RT treatment, it significantly induced G_2_/M arrest and cell death. These data show that dactolisib arrests the cell cycle at the G_2_/M phase after radiation-induced DNA damage in addition to blocking effects at the G_1_/S transition.

In addition to PI3K pathway activation, inactivation of the p27 tumor suppressor plays a critical role in tumor progression [28]. P27 is an important mediator of G1/S cell-cycle arrest that is rarely mutated in solid cancers [29]. As the p27 pathway is often impaired in GBM, it is regarded a tumor suppressor [30].

In this study, we found that p27 plays a role in dactolisib-mediated enhancement of cytotoxicity in glioma cells cotreated with TMZ and RT, as indicated by its higher expression under TMZ+RT+dactolisib as revealed by AKT/PKB Phospho Antibody Array analysis. Migration and invasion are important aspects of tumor progression and are closely associated with cancer recurrence and therapeutic resistance in GBM [31]. This study showed that TMZ+RT+dactolisib effectively inhibited the ability of glioma cell lines to grow, migrate, and invade, as well as increased radio sensitivity. This suggests that dactolisib exerts broad anti-tumor activity and potently enhances the effects of TMZ+RT.

Bcl-2 family proteins include the anti-apoptotic agent Bcl-2 and the pro-apoptotic agent Bax. The apoptotic pathway is under tight regulation of Bcl-2 proteins [32, 33]. In this study, we found that Bcl-2 plays an important role in the mechanism of dactolisib in enhancing TMZ+RT efficacy, as it was more strongly downregulated in the TMZ+RT+dactolisib group than in the TMZ+RT group. Dactolisib enhanced the downregulation of Bcl-2, which indicates that it improves treatment efficacy in part by promoting cancer cell apoptosis. The PI3K-AKT pathway regulates cell survival signals to induce cell growth and to hinder cell death. PI3K-AKT signaling may mediate anti-apoptotic activities by changing the ratio of pro- and anti-apoptotic proteins [6, 26].

Results of the AKT/PKB Phospho Antibody Array analysis of SHG44 cells showed that p-AKT levels decreased after TMZ+RT+dactolisib treatment in all three cell lines; p-AKT is involved in resistance against TMZ and inhibition of cell migration and invasion. Furthermore, treatment of cells with TMZ+RT+dactolisib resulted in better outcomes than the combination of any two of the three treatment modalities tested.

The *in-vitro* assessment in this study showed that dactolisib exerts potent cytotoxic activity and sensitizes GBM to TMZ+RT. The results of this study corroborate with recent findings indicating that PI3K inhibition is enhanced with RT therapy. Based on the prolonged survival time and inhibited glioma growth in a nude rat model, we confirmed that dactolisib improved the therapeutic effect of TMZ+RT. Currently, there are no ongoing clinical studies on the use of dactolisib in GBM. However, we previously observed side effects of dactolisib in rats, which lack emetic reflex. It is reported that by adding dihydrotestosterone, the side effects of dactolisib could be reduced in breast cancer mice [34]. Future studies should aim at overcoming/reducing the side effects of therapeutic drugs, while further strengthening their antitumor effects. MRI was used to evaluate glioma progression and volume. This technique provides high-quality images and is convenient for usage as it is non-invasive and allows continuous monitoring of tumor progression. MRI should be the gold standard for evaluating glioma in animal experiments.

In conclusion, our data on dactolisib anti-tumor efficacy obtained in *in-vitro* and *in-vivo* experiments suggest that the PI3K/mTOR inhibitor dactolisib can enhance the cytotoxicity of TMZ+RT treatment. Dactolisib is a treatment option in addition to the current SOC therapy for GBM to improve SOC therapy efficacy.

## MATERIALS AND METHODS

### Cell culture

The A172 and SHG44 glioma cell lines were purchased from the Chinese Academy of Sciences, Shanghai, China. The human GBM cell line, T98G, was obtained from the American Type Culture Collection. A172 cells were maintained in Dulbecco’s Modified Eagle’s Medium (DMEM, Gibco) supplemented with 1% penicillin/streptomycin (Invitrogen) and 10% fetal bovine serum (FBS, Gibco). SHG44 cells were cultured in RPMI-1640 medium (Gibco) supplemented with 10% FBS. T98G cells were maintained in MEM containing 1% penicillin/streptomycin, 1% NEAA, and 10% FBS.

### Radiation treatment

An MBR-1520R-3 irradiator (Hitachi, Japan) was used for irradiating cells and rats. Cultured cells and rats were irradiated with X-rays (2 Gy).

### Cell viability assay

Cell viability was assessed using a cell counting kit-8 (CCK-8) (Dojindo, Japan) assay, per the manufacturer’s protocol. Cells were plated in 96-well plates at 2–5 × 10^3^ cells/well and allowed to adhere for 24 h at 37°C. The cells were left untreated for 24 h or treated with 100 µM TMZ and different concentrations of dactolisib in the presence of radiation for 2 h after TMZ. Then, CCK-8 reagent (10 µL) was added to each well, and plates were incubated for 2–4 h at 37°C. Optical density (OD) was measured at 450 nm using an Elisa plate reader (Thermo Scientific Multiskan GO, Finland). Cell viability was expressed as a percentage of the untreated control.

### Cell migration assay

Cell migration was assayed by wound closure assay (Corning, Rochester, NY, USA). Cells were cultured in 6-well plates and serum-starved for 12 h, and then, each well was divided into a 2-part grid. The monolayer was scratched in a straight line by using a 1-ml pipette tip and the debris was removed. Twenty-four hours later, each well was photographed at 20× magnification. Gap-closure areas were measured using the ImageJ software.

### Cell invasion assay

Cell invasion was measured using the Transwell system (Corning). The invasion of GBM cells was tested in a 24-well transwell plate including invasion chambers with an 8-µm pore-size polycarbonate membrane, with or without Matrigel coating. Cells were trypsinized, resuspended, and counted. Cells (10^4^ cells/well) were seeded in the upper chamber in starvation medium. After 24-h incubation, the inserts were fixed methanol and stained with 1% crystal violet. The cells on upper surface of the chamber were removed using a cotton swab, and the invaded cells on lower membrane surface were counted.

### Cell cycle assay

The different cell lines were respectively plated in 6-well cell culture plates. After 48 h of incubation in the presence or absence of dactolisib at room temperature, the cells were collected and analyzed by flow cytometry (FACSCanto II; BD Biosciences) using a CycleTEST^™^ PLUS DNA Reagent Kit (BD Biosciences, USA), according to the manufacturer’s protocol. The data obtained were analyzed using ModFit software.

### Apoptosis assay

Apoptotic cells were quantified using an Annexin V apoptosis detection kit (BD Biosciences). Cells at the logarithmic growth phase were seeded in 6-well plates at 2 × 10^5^ cells/well. Cells from each treatment group were harvested with Accutase detachment solution (Sigma-Aldrich, USA) and labeled with Annexin-V-FITC/PI (BD Biosciences) following the manufacturer’s instructions. The stained cells were analyzed by flow cytometry. The number and percentage of necrotic (annexin V+/PI+), apoptotic (Annexin V+/PI−), and viable (annexin V−/PI−) cells were calculated using the FACSDiva software (Version 6.2).

### Western blotting

In total, 60 μg of protein was loaded onto a 10% sodium dodecyl sulphate-polyacrylamide gel and resolved by electrophoresis at 100 V for 2 h. After the proteins were transferred to a polyvinyldifluoridene membrane, the membrane was blocked in 3% nonfat milk in Tris-buffered saline/Tween-20 (0.5%) (TBS/T) for 40 min at 37°C and then incubated with rabbit polyclonal antibodies against mouse p-Akt^S473^, MGMT, Bcl-2, and p27 (Santa Cruz, CA, USA), each of which was used at a 1:500 dilution in TBS/T, overnight at 4°C. After 3 washes with TBS/T, the membranes were incubated with HRP-conjugated anti-rabbit IgG in TBS/T (1:2,500) for 45 min at 37°C. The immunoreactive bands were visualized with an enhanced chemiluminescence kit and captured on X-ray film.

### Phosphorylation analysis

The Phospho Explorer Antibody Array (PAB216; Full Moon Biosystems, Sunnyvale, CA, USA) was used per the manufacturer’s instructions. Briefly, samples were obtained and scanned on a chip scanner (GenePix 4000B; Axon Instruments, USA). The results were analyzed with GenePix Pro software.

### Rat glioma model

Rats were kept in a pathogen-free environment at constant temperature and humidity. Rats were placed in a stereotactic frame and anesthetized using isoflurane. SHG44 cells (3 × 10^5^ in 10 μL PBS) were injected into the right caudate nuclei of immunodeficient rats (GaoGe, Shanghai, China), 3 mm lateral from the midline, 1 mm posterior, and at 5 mm depth from the bregma, using a 25-μM syringe. The rats were observed daily and treatment was initiated 4 days later, after successful establishment of the glioma was verified through magnetic resonance imaging (MRI). The rats were divided into three groups of 8 randomly. One group received TMZ+RT+dactolisib, one TMZ+RT, and one group was left untreated as a control. Tumor volumes were examined with MRI at days 7, 10, and 14 days after injection. The rats were weighed and clinical symptoms were observed every day. The rats were anesthetized with an intraperitoneal injection of chloral hydrate (300 μL/100 g). The rats were sacrificed by cervical dislocation after moribund. Surviving rats were sacrificed at day 30.

### Statistical analysis

Data are expressed as the mean ± standard deviation (SD). *P*-values < 0.05 were considered statistically significant. All statistical analyses were performed using GraphPad Prism 7.0 software.

## SUPPLEMENTARY MATERIALS FIGURE


