# Correction: The dual PI3K/mTOR inhibitor dactolisib elicits anti-tumor activity *in vitro* and *in vivo*

**DOI:** 10.18632/oncotarget.25063

**Published:** 2018-03-30

**Authors:** Fei Shi, Jinying Zhang, Hongyu liu, Liangliang Wu, Hongyu Jiang, Qiyan Wu, Tianyi Liu, Meiqing Lou, Hao Wu

**Affiliations:** ^1^ Department of Neurosurgery, Shanghai General Hospital, Shanghai Jiao Tong University School of Medicine, Shanghai 200000, China; ^2^ Institute of Basic Medicine Science, Chinese PLA General Hospital, Beijing 100853, China; ^3^ Key Laboratory of Cancer Center, Chinese PLA General Hospital, Beijing 100853, China; ^4^ Department of Anesthesiology, Wuxi Third People's Hospital, Wuxi, Jiangsu 214000, China; ^5^ Department of Neurosurgery, Xuanwu Hospital, Capital Medical University, Beijing 100053, China

**This article has been corrected:** Dr. Meiqing Lou is included as a co-corresponding author.

Original article: Oncotarget. 2018; 9:706-717. https://doi.org/10.18632/oncotarget.23091

