# Supplementary material for: The dual PI3K/mTOR inhibitor dactolisib elicits anti-tumor activity in vitro and in vivo
Source: Oncotarget. 2017 Dec 9;9(1):706–17. doi: 10.18632/oncotarget.23091 (PMC5787502; doi:10.18632/oncotarget.23091)
Supplement: Supplementary file 1 [file oncotarget-09-706-s001.pdf]

The dual PI3K/mTOR inhibitor dactolisib elicits anti-tumor activity *in vitro* and *in vivo*

SUPPLEMENTARY MATERIALS

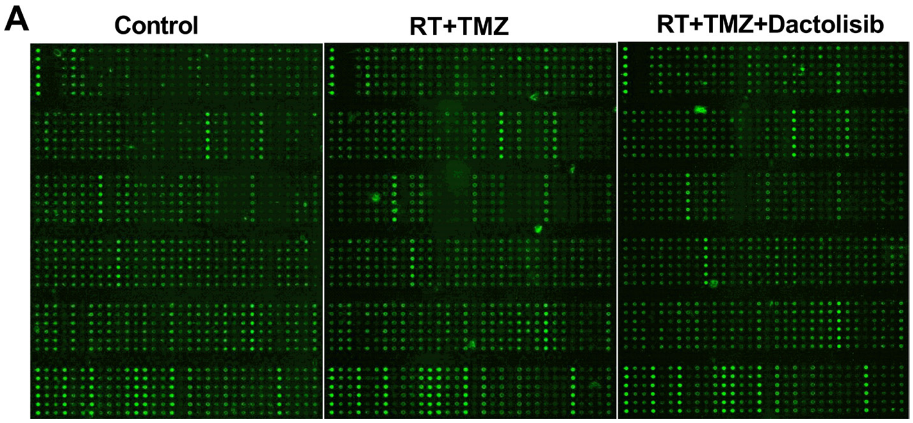

Supplementary Figure 1: Anti-tumor and pro-apoptotic effects of dactolisib are associated with a reduction of p-AKT and mTOR. SHG44 cells were treated with TMZ+RT or TMZ+RT+Dactolisib and subjected to microarray analysis. Non-treated SHG44 cells were used as a control. Compared to TMZ+RT, treatment with TMZ+RT+dactolisib induced overexpression of p27 and decreased p-AKT, mTOR, and Bcl-2 protein levels.
